# Programmable Stepwise Heteroepitaxial Growth of Colloidal Crystals With Different Phases

**DOI:** 10.1002/adma.73583

**Published:** 2026-06-03

**Authors:** Xiaowei Liu, Yuanwei Li, Ramin Yazdaanpanah, Ye Zhang, Rachel R. Chan, Xiaobing Hu, Yiming Yang, Vinayak P. Dravid, Koray Aydin, Chad A. Mirkin

**Affiliations:** ^1^ Department of Materials Science and Engineering Northwestern University Evanston Illinois USA; ^2^ International Institute for Nanotechnology Northwestern University Evanston Illinois USA; ^3^ Department of Chemical and Biological Engineering Northwestern University Evanston Illinois USA; ^4^ Department of Electrical and Computer Engineering Northwestern University Evanston Illinois USA; ^5^ Department of Chemistry Northwestern University Evanston Illinois USA

**Keywords:** colloidal crystals, DNA, fcc‐bcc heterointerface, heteroepitaxial growth, lattice mismatch, nanomaterials

## Abstract

Heteroepitaxial growth is a powerful strategy for constructing hierarchical systems by integrating materials with different structures across the angstrom to nanometer length scale. However, lattice mismatches between different phases often impact the resulting crystal stability. This is especially true for colloidal crystal systems. Here, colloidal crystal engineering with DNA is used to assemble multi‐phase colloidal crystals, with extreme tolerance for lattice strain. Most notably, the structural flexibility of DNA can accommodate lattice mismatch up to 18%, allowing one to grow, for the first time, face‐centered cubic (fcc) lattices with (111) facets on body‐centered cubic (bcc) crystals with (110) facets (a 13% bcc‐fcc phase misfit for the particles studied; 2%–4% in an atomic system). By adjusting particle size, more or less strain can be induced, allowing one to determine the upper limit for bcc‐fcc phase misfit (34%). Finite‐difference time‐domain (FDTD) optical simulations reveal that these multi‐phase heteroepitaxial structures can function as waveguides, making them attractive targets for those interested in optics. The lattice mismatches accommodated through DNA bonding exceed those typical in atomic heteroepitaxy (a few percent without a buffer layer), highlighting the versatility of this technique for designing and preparing hierarchical materials with tailored structure‐function relationships.

## Graphical abstract

Stepwise heteroepitaxial growth is adapted to colloidal crystal engineering with DNA, enabling face‐centered cubic (fcc) facets to grow on body‐centered cubic (bcc) crystals with 110 facets. This approach tolerates large lattice mismatches, extending heteroepitaxy beyond the limits of conventional atomic systems.

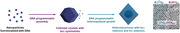

## 1 Introduction

The development of modern microelectronic and photonic devices hinges on the ability to grow single‐crystalline materials on substrates with differing compositions and crystal structures, a process known as heteroepitaxial growth [1, 2, 3, 4]. Heteroepitaxy enables the integration of disparate materials into multi‐material devices with superior performances and emergent properties that surpass those of the individual building blocks [5, 6, 7]; such systems can engage in interesting structural transformations [8], band bending processes [9], and facet‐selective catalytic reactions [10], thereby expanding the potential of many next‐generation technologies. However, lattice mismatches between materials with different crystal structures can create system strain [11, 12], so the diversity of materials that can be integrated through heteroepitaxial growth is restricted [13, 14]. In colloidal crystal systems, nanoparticle size dispersity also complicates epitaxial integration. Unlike atomic systems, where precise, stepwise growth can be achieved using techniques such as physical and chemical vapor deposition [15, 16], analogous techniques do not currently exist in colloidal systems. As a result, previous attempts at preparing colloidal crystals via heteroepitaxial growth processes have typically only yielded crystals with discontinuous islands of a few ordered particle layers [17, 18, 19]. These limitations highlight the need for new strategies that enable controlled epitaxial growth in colloidal systems [20].

Colloidal crystal engineering with DNA is one of the most versatile tools for synthesizing ordered nanoparticle superlattices [21, 22, 23]. Nanoparticles with diverse sizes and shapes, densely functionalized with DNA strands, can be assembled into ordered, three‐dimensional superlattices through Watson–Crick base pairing [24, 25]. This approach has enabled the construction of crystals with complex phases and tunable properties (over 100 symmetries to date) [21, 26, 27]. Compared to other types of interparticle linkers used in colloidal crystal assembly, DNA has several key advantages: DNA bonds can accommodate elastic strains of up to 7.7% [28]; tolerate large lattice deformations [29]; and enable stepwise seed‐mediated growth [30]. The epitaxial growth of crystals has been demonstrated using colloidal crystal engineering with DNA, although it has largely been used in disordered [31] or single‐phase systems [28, 30]. Achieving well‐defined, multi‐phase 3D colloidal crystals via heteroepitaxial growth remains challenging due to the large lattice mismatches at phase boundaries, which create instability and impede directional growth.

Herein, we present a strategy for using DNA‐programmable heteroepitaxial growth to yield multi‐phase colloidal crystals. Specifically, a DNA design that enables programmable and controlled growth of particles in a face‐centered cubic (fcc) lattice on particles in a body‐centered cubic (bcc) lattice is proposed and explored. Experimental results and theoretical modeling reveal that the (111) plane of fcc crystals preferentially grows on the (110) facet of the bcc crystals, primarily because the lattice mismatch of around 18% at the interface is effectively mitigated by DNA bond flexibility. In these multi‐phase colloidal crystals, the bcc‐fcc phase misfit at the heterointerface is around 13%, significantly exceeding that observed in atomic bcc–fcc transitions (2%–4%) [32]. Furthermore, finite‐difference time‐domain (FDTD) simulations indicate that the phase heterogeneity of the as‐synthesized heterostructures could be useful in devices that rely on the manipulation of light absorption. This approach is generalizable to a broad range of phase combinations and building block sizes, including those with larger bcc‐fcc phase misfits up to 34%. As such, it should allow for the controlled integration of materials within heterostructured colloidal crystals in ways that unlock unique optical, mechanical properties.

## 2 Results

**FIGURE 1:**
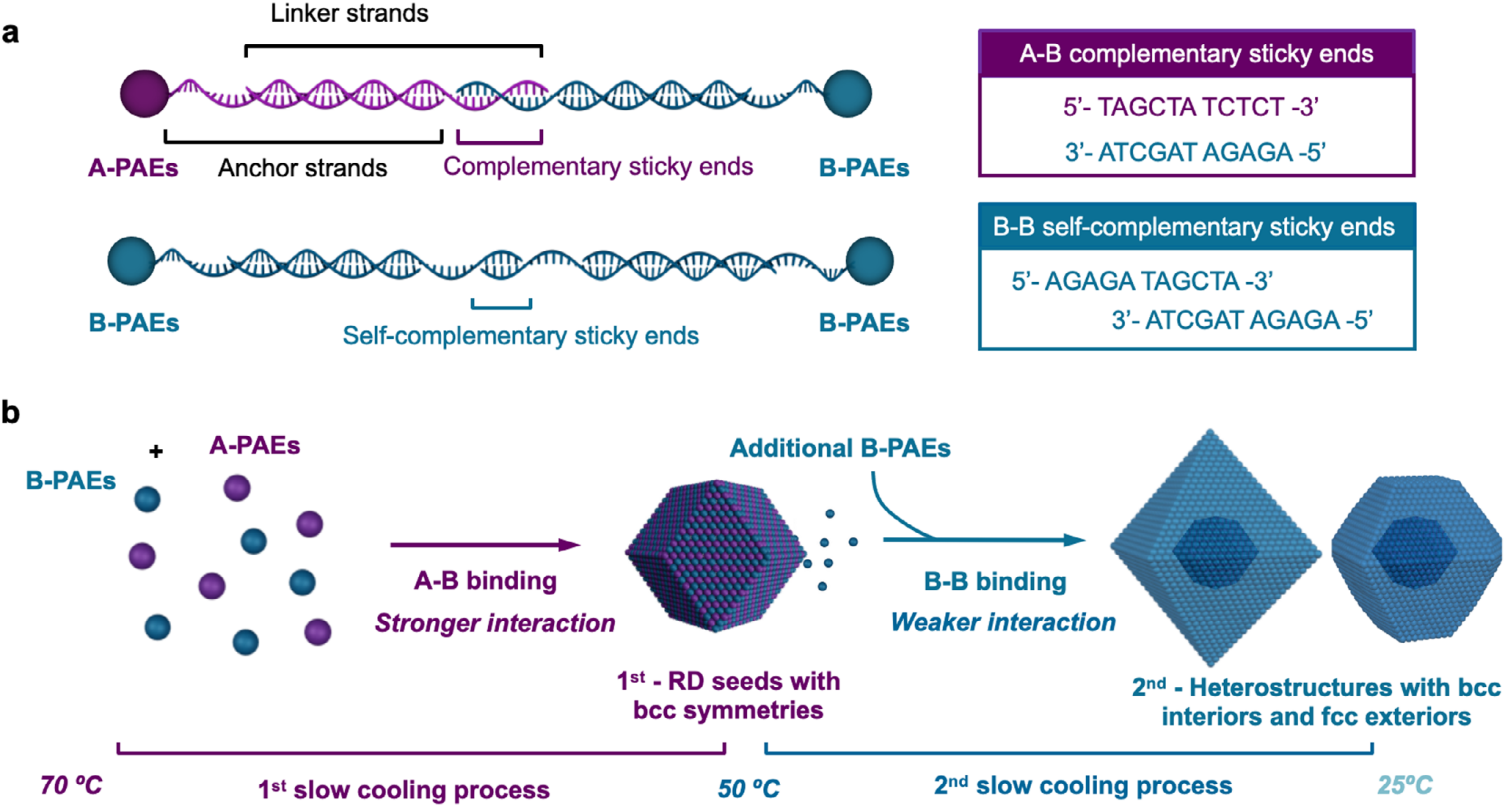
DNA design for stepwise heteroepitaxial growth of colloidal crystals with regions that have different phases. (a) Schematic of A and B‐type PAE structures. The complementary binding between A and B PAEs is based on 11 DNA base pairing interactions (complementary sticky ends), and the self‐complementary binding between B PAEs is based on six base pairs contained in the 11‐base sticky end. (b) A synthesis scheme of the heteroepitaxial growth process of a colloidal crystal. The bcc seeds (A‐B PAEs) nucleate first due to their stronger sticky‐end interactions, followed by the heteroepitaxial growth of additional B PAEs (fcc structures) driven by weaker sticky‐end interactions. The final heterostructures are expected to exhibit octahedral or truncated‐octahedral shapes derived from the fcc structures, with rhombic dodecahedral bcc seeds encapsulated inside.

Two different types of spherical nucleic acid (SNA) nanoparticle conjugates [23, 26], each composed of 20 nm gold nanoparticle cores densely functionalized with DNA shells of different sequences, were synthesized. These structures, designated as ‘A’ or ‘B’ are “programmable atom equivalents” (PAEs) [21, 33, 34], where the nanoparticle core represents an “atom” and the DNA hybridization interactions between the strands on the shell serve as “the bonding elements.” Their DNA‐mediated assembly is dictated by the design rules of colloidal crystal engineering with DNA and the complementary contact model (CCM) [35, 36], where thermodynamically favored structures are those that arrange particles in such a way as to maximize hybridization or bond formation. The sticky ends (short single‐stranded overhangs at the periphery of the DNA shell) were designed such that the ‘A’ and ‘B’ PAEs form 11 base pair (bp) complementary hybridization interactions, resulting in the formation of bcc colloidal crystal structures [37]. The ‘B’ PAEs were designed to hybridize and form fcc crystal structures through their five‐bp self‐complementary sticky ends (Figure 1a, Table S1). Notably, the 11‐bp ‘A’ PAE‐‘B’ PAE interactions (annealing temperature *T*_a1_ = ∼60°C) are stronger than the six‐bp ‘B’ PAE‐‘B’ PAE self‐complementary interactions (annealing temperature *T*_a2_ = ∼35°C) (Table S2). Consequently, as the system is slowly cooled, the ‘A’ PAE‐‘B’ PAE interaction occurs first at higher temperatures (i.e., the bcc seed structure is formed first).

This DNA design enables stepwise growth of ‘A’ and ‘B’ PAEs as the mixture of them is slow‐cooled from 70°C to 25°C (0.01°C per minute), driven by the difference in binding strength of the ‘A’‐‘B’ and ‘B’‐‘B’ interactions (Figure 1b). This controlled growth process is expected to yield heterostructured colloidal crystals with multiple phases (Figure 1b). In the first stage of slow‐cooling (70°C–50°C), the stronger ‘A’ and ‘B’ interactions (1:1 ‘A’‐‘B’ ratio) will lead to the formation of ‘seed’ bcc colloidal crystals with rhombic dodecahedron (RD) habits at equilibrium. In the second stage (50°C–25°C), when additional ‘B’ PAEs are added to the solution, self‐hybridization of ‘B’ PAEs will occur on the surface of the ‘A’‐‘B’ bcc seeds. The exposed facets of the bcc crystals act as substrates for the stepwise growth of ‘B’ PAEs into fcc superlattices, resulting in octahedron and truncated octahedron crystal habits extending from the RD bcc crystals. To promote complete overgrowth and encapsulation of the bcc seeds by fcc structures, the amount of B PAEs introduced in the second step was increased to six times those used in the first step experimentally.

**FIGURE 2:**
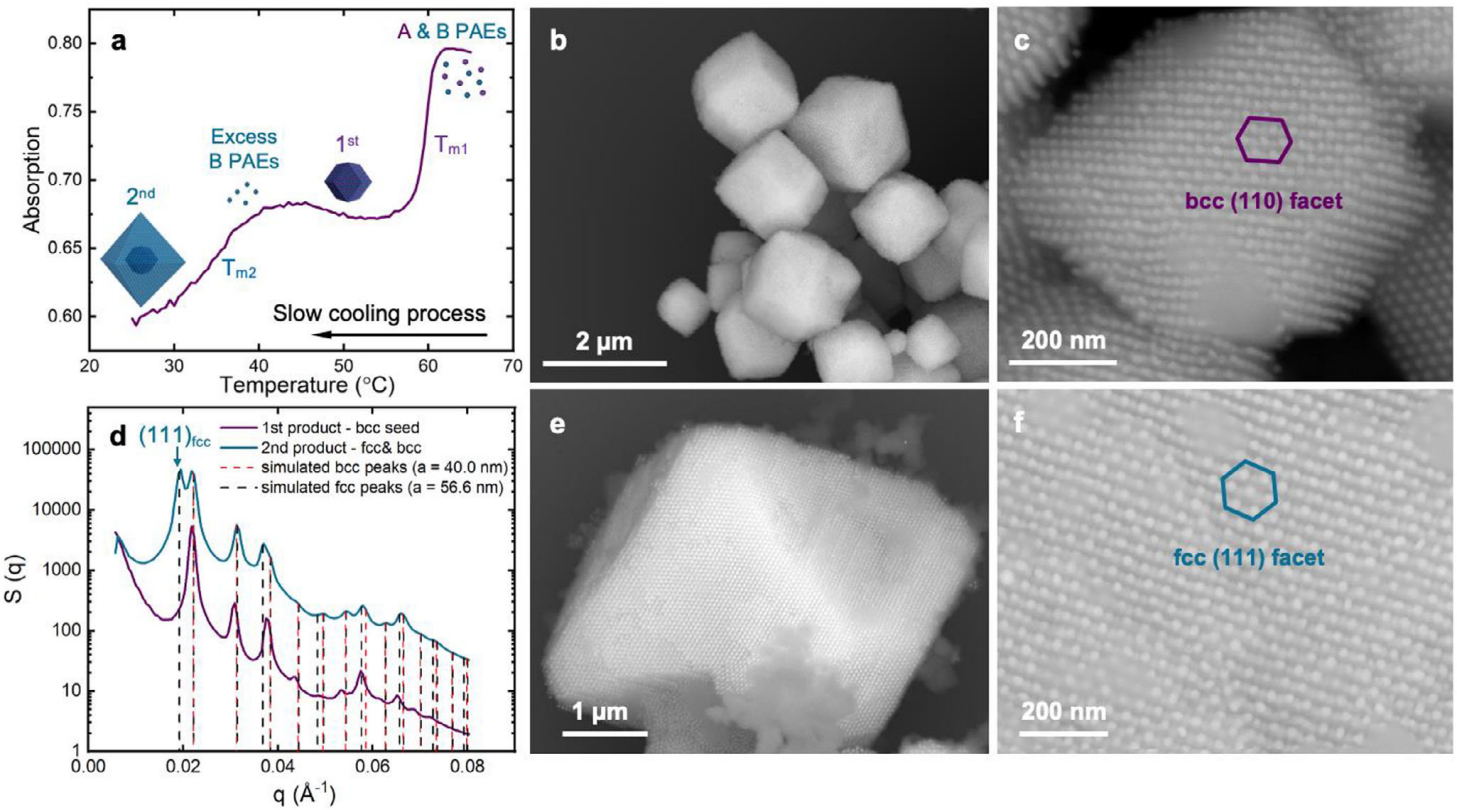
Characterization of the products with different structures and symmetries for each phase of crystal growth. (a) In situ variable‐temperature UV–vis spectroscopy of the PAE assembly process (at 520 nm). Two distinct annealing transitions are observed during the slow‐cooling process. (b,c) SEM images of the bcc products present after the first annealing transition. (d). SAXS characterization of the products after each annealing transition in the slow‐cooling process. The SAXS signal of the solution after the first annealing transition indicates the presence of structures with bcc symmetries, while after the second annealing transition, peaks indicating the presence of crystals with fcc and bcc symmetries are observed. (e,f) SEM images of the products of the second annealing transition showing the presence of fcc superlattice materials.

This growth pathway for the heterostructures with fcc and bcc phases was elucidated by performing in situ variable‐temperature UV–vis absorbance measurements on mixtures of ‘A’ and ‘B’ PAEs during slow‐cooling (Figure 2a). The absorbance of the PAE solution at 520 nm (the maximum extinction of the dispersed 20 nm gold nanoparticle PAEs in solution) was monitored. A decrease in absorbance accompanied by a redshift in extinction peak was observed as the PAEs assembled into crystals in solution. Two distinct transitions were observed, each corresponding to a separate annealing transition. The first transition, occurred at ∼60°C (T_a1_), consistent with the stronger ‘A’‐‘B’ complementary interactions, while the second transition occurred at ∼35°C (T_a2_), indicating the formation of weaker ‘B’‐‘B’ self‐complementary interactions.

The assembled products formed after each annealing transition were characterized using small‐angle X‐ray scattering (SAXS) and scanning electron microscopy (SEM). As expected, the SAXS patterns of the 1st products formed through ‘A’‐‘B’ complementary sticky‐end hybridization closely matched the simulated patterns for bcc PAE arrangements with a lattice constant of approximately 40.0 nm (Figure 2d, Table S3). SEM images confirmed that these bcc seeds exhibited characteristic rhombic dodecahedral (RD) morphologies and exposed (110) facets (Figure 2b,c, Figure S1). The SAXS signal of the 2nd colloidal crystal products formed after the second annealing transition displayed peaks corresponding to both the bcc seeds as well as crystal structures with fcc symmetry and a lattice constant of approximately 56.6 nm (Figure 2d). SEM images revealed that the resultant crystals had truncated octahedral or octahedral shapes, characteristic of fcc structures, primarily with (111) facets exposed (Figure 2e,f, Figure S2, Table S4). The dominant products after the second step are overgrown heterostructures, with no clearly detectable population of standalone bcc seeds. This result indicates that during the second growth step, fcc growth preferentially occurred upon bcc seeds, forming heterostructures with RD cores and extended octahedral or truncated‐octahedral shells (Figure S2d). This observation may be attributed to the role of the pre‐existing bcc seeds as templates, which substantially lowers the free‐energy barrier for fcc nucleation.

**FIGURE 3:**
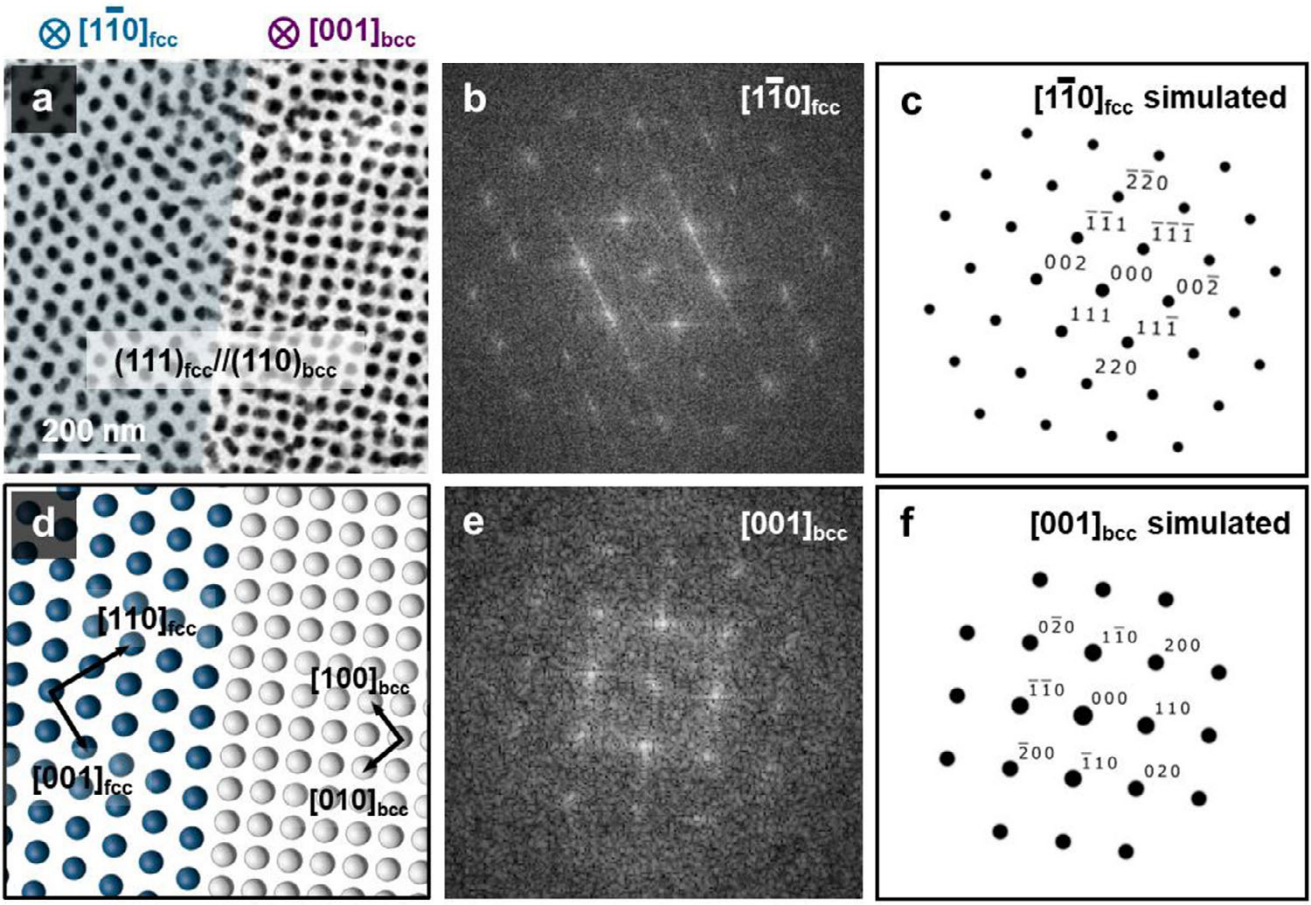
Structural characterization of the bcc‐fcc interface (a) Annular bright field (ABF) image of the phase heterogeneity of bcc (shaded gray) and fcc (shaded blue) structures. (b) Digital FFT pattern of blue area of a. (c) The corresponding diffraction simulation of fcc structure along the $\left[1\bar{1}0\right]$_fcc_ zone‐axis. (d) Schematic for the interface between fcc and bcc superlattices. (e) Digital FFT pattern of gray area of a. (f) The corresponding diffraction simulation of the bcc structure along the [001]_bcc_ zone‐axis.

To better understand the heteroepitaxial growth of fcc crystal structures on bcc seeds at high spatial resolution, cross‐sections of the heterostructured crystals were analyzed using transmission electron microscopy (TEM). Distinct interfaces were observed between the two different crystal phases (Figure S3). Specifically, the projections of the PAE crystals viewed along the perfect crystal zone axes indicate that one region closely resembles an fcc structure projected along the $\left[1\bar{1}0\right]$_fcc_ direction (Figure 3a, blue color shaded area), while the other region corresponds to a bcc structure oriented along the [001]_bcc_ direction (Figure 3a). A comparison of the digital fast Fourier transform (FFT) patterns from each region of the crystal (Figure 3b,e) with the corresponding simulated diffraction patterns (Figure 3c,f) confirms the assigned crystallographic orientations and lattice symmetries of both phases. More specifically, the FFT of the left side of the interface (Figure 3b) matches the simulated diffraction patterns of single‐crystalline fcc structures projected along the $\left[1\bar{1}0\right]$_fcc_ direction (Figure 3c), while the FFT from the right side of the interface (Figure 3e) aligns with the simulated projection of bcc structures along the [001]_bcc_ direction (Figure 3f), further supporting the identification of the bcc‐fcc heterointerface.

**FIGURE 4:**
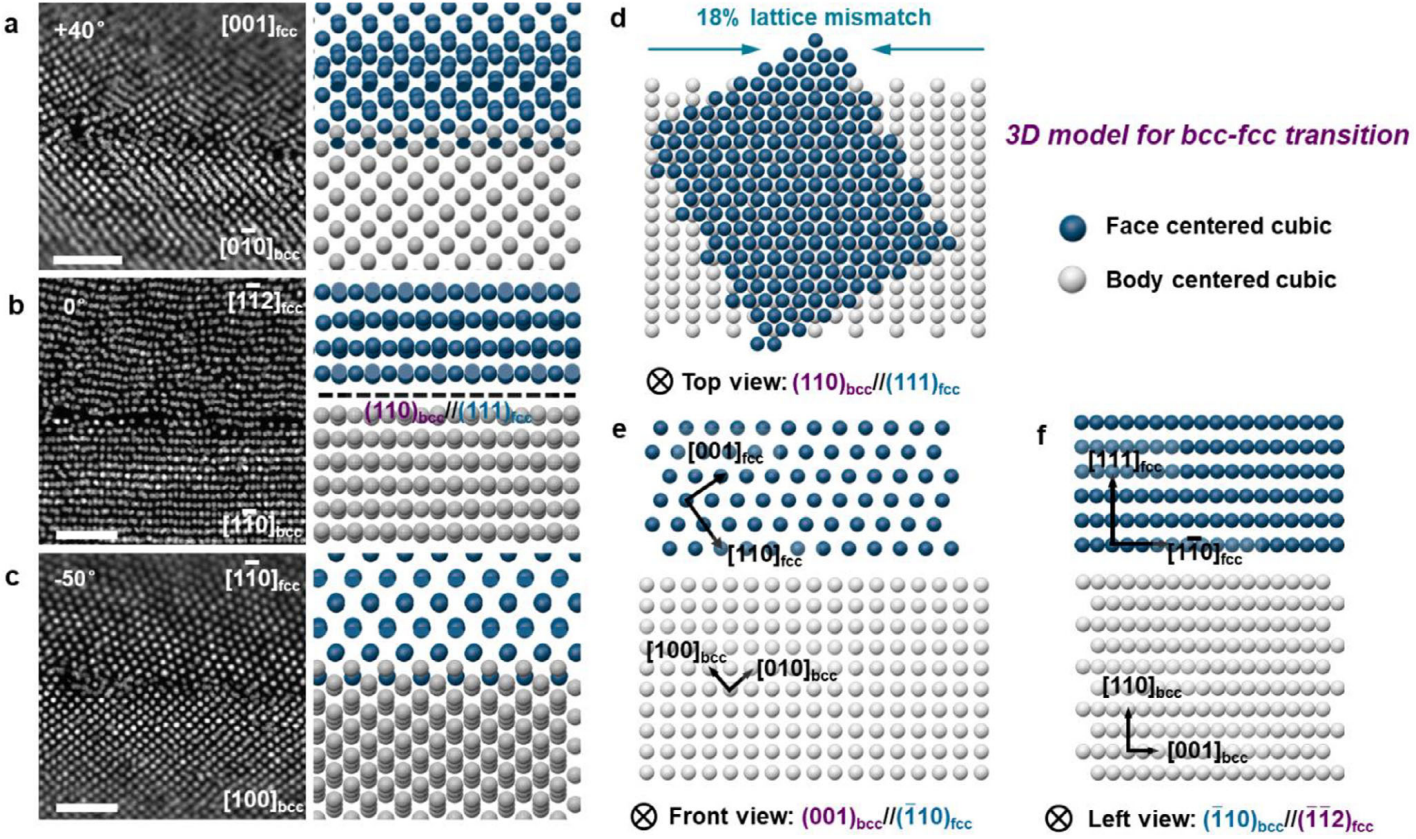
Characterization of the interfacial region between fcc and bcc structures at different tilting angles. (a–c), Left panel: TEM images of the interface between fcc and bcc lattices viewed at different tilting angles: +40°, 0°, and ‐50°, right panel: the matching projections from the reconstruction model. Scale bars, 200 nm. (d–f), Top view, front view, and left view of the interface between fcc and bcc from 3D reconstruction model based on serial TEM tilting characterizations.

To visualize the internal structure at the bcc‐fcc assembly, we performed a series of tilting experiments. The experiments revealed how the crystallographic orientations of the two phases evolve with tilt angles, with the tilt axis lying approximately parallel to the transition interface (Figure 4a–c, left panels, and Figure S4). When the fcc domain is oriented along the $\left[1\bar{1}2\right]$_fcc_ zone‐axis, the bcc domain aligns precisely along the $\left[1\bar{1}0\right]$_bcc_ zone‐axis. Tilting the sample by +40° rotates the fcc structure toward ∼[001]_fcc_ zone‐axis, corresponding to approximately the $\left[0\bar{1}0\right]$_bcc_ axis in the bcc structure. Tilting by ‐50° rotates the fcc structure toward $∼[1\bar{1}0]$_fcc_, which corresponds to roughly the [100]_bcc_ zone‐axis. These observations establish the crystallographic relationships between the two lattices and clarify how the fcc phase grows epitaxially on bcc seeds in colloidal crystals. Projections from the reconstructed structural model match the experimental images when one disordered interfacial layer is removed to enhance clarity (Figure 4a–c, right panels). From the top‐view of the reconstruction (Figure 4d), the common interface plane is identified as (110)_bcc_//(111)_fcc_. In the front view (Figure 4e), the [001]_bcc_ axis aligns with $\left[\bar{1}10\right]$_fcc_ axis, and in the left view (Figure 4f), the $\left[\bar{1}10\right]$_bcc_ direction aligns with the $\left[\bar{1}\bar{1}2\right]$_fcc_. This confirms that the (111)_fcc_ plane is the preferred stacking and growth plane on the exposed (110)_bcc_ facets during this stepwise assembly process (Figure S5). The interplanar spacings of the $\left(\bar{1}10\right)$_fcc_ and $\left(\bar{1}\bar{1}2\right)$_fcc_ planes are 40.0 and 23.1 nm, respectively, whereas the interplanar distance of (001)_bcc_ and $\left(\bar{1}10\right)$_bcc_ planes were measured as 40.0 and 28.3 nm, respectively (Table S5). As a result, the lattice mismatch between (001)_bcc_ and $\left(\bar{1}10\right)$_fcc_ is nearly zero, whereas the mismatch between $\left(\bar{1}10\right)$_bcc_ and $\left(\bar{1}\bar{1}2\right)$_fcc_ reaches 18%, calculated using *f*  = |*d_fcc_* − *d_bcc_* |/*d_bcc_* . To our knowledge, this represents the largest lattice mismatch reported in a colloidal crystal system. The flexible DNA “bonds” accommodate the resulting strain [28, 29]. This phenomenon has been explained through a theoretical model and analysis in (Equations S1–S5). More broadly, these findings demonstrate that this assembly strategy tolerates lattice mismatches far exceeding those permissible in atomic epitaxy, where mismatches of only a few percent require buffer layers [1].

This derived configuration closely resembles the well‐established model (Nishiyama–Wassermann orientation relationship) for bcc‐to‐fcc transitions in atomic systems [38, 39]. In both cases, the common interface plane between the two phases is (110)_bcc_ //(111)_fcc_. In atomic systems, atoms adopt this configuration to minimize the energy generated by lattice mismatch between the fcc and bcc phases, allowing the bcc structures to nucleate and grow on fcc templates and driving phase transformation. Similarly, in the DNA‐mediated stepwise growth process, adopting this orientation effectively eliminates the lattice mismatch along [001]_bcc_//$\left[\bar{1}10\right]$_fcc_, thereby reducing the energy barrier for heteroepitaxial growth of fcc structures on bcc ones. To quantify the phase differences between fcc and bcc phases during heteroepitaxial growth, we introduce the bcc‐fcc phase misfit factor, defined as $\delta_{fcc/bcc}\equiv \left|a_{fcc}-\sqrt{3/2}a_{bcc}\right|/a_{fcc}$, where *a_fcc_* and *a_bcc_* represent the lattice constants of the fcc and bcc structures, respectively [32]. The bcc‐fcc phase misfit factor observed between bcc and fcc phases in our model is approximately 13%, which is significantly larger than the values observed at bcc‐fcc heterointerfaces in atomic systems, ranging from 2%–4% in pure iron and steels [40, 41], and approximately 3% between bcc iron and fcc copper [42].

**FIGURE 5:**
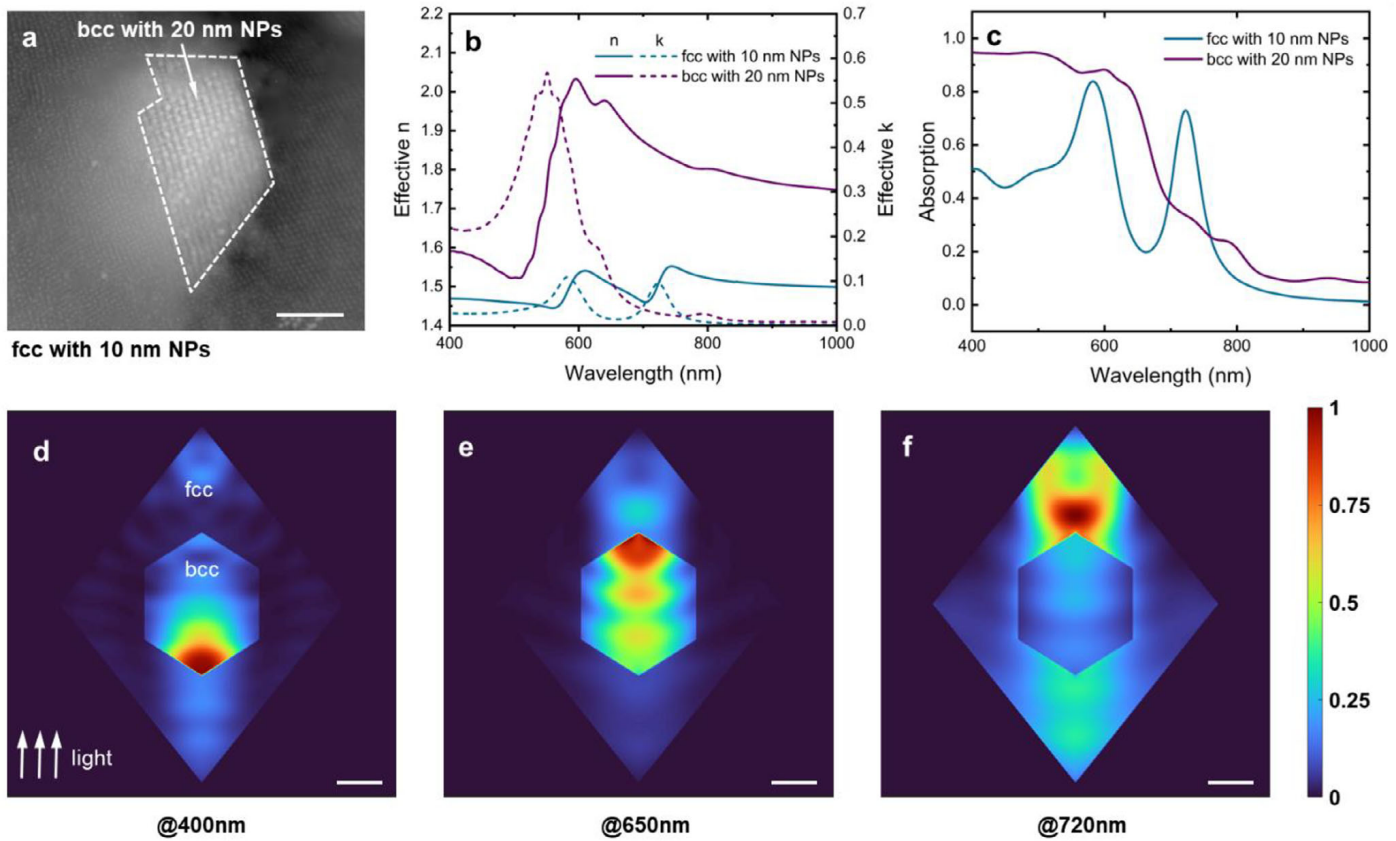
Optical property simulations of heterogeneous colloidal crystals. (a) SEM images of heteroepitaxially grown crystals. Note that the bcc phases were constructed using 20 nm gold nanoparticle PAEs and the fcc phases were grown using 10 nm gold nanoparticle PAEs. (b,c) Simulated effective index and absorption spectra for the two distinct phases. (d–g) Simulated normalized absorption mapping for heterostructured crystals with two phases, as the direction of light illumination is from bottom to up. Scale bars, 200 nm.

Furthermore, this method can be readily extended to other systems (Figure S6), including those involving PAEs with different hydrodynamic radii, where the bcc‐fcc phase misfit factor may exceed 13%, highlighting the great potential of this approach for engineering heteroepitaxy within multi‐phase colloidal crystals. For example, multi‐phase colloidal crystals were prepared via the heteroepitaxial growth of fcc lattices of 20 nm PAEs on bcc crystals comprised of 10 nm PAEs (Figures S7 and S8). In these multi‐phase structures, the bcc‐fcc phase misfit factor (δ_fcc⁄bcc_) can be as high as 34%. In addition, the structural symmetry between core and shell can be reversed: heterostructured colloidal crystals could be prepared via the heteroepitaxial growth of fcc lattices of 10 nm PAEs on bcc seeds formed from 20 nm PAEs (Figure 5a, Figures S9 and S10), with a corresponding bcc‐fcc phase misfit factor (δ_fcc⁄bcc_) of 19%. These results highlight the versatility of this approach for achieving heteroepitaxial growth, suggesting that DNA‐mediated assembly can tolerate significant bcc‐fcc misfits, given the differences in both phase and size of building blocks (Table S4).

The optical properties of the multi‐phase colloidal crystals were investigated using FDTD simulations. Specifically, the analysis focused on heterostructures synthesized using 20 nm PAEs for the bcc phase (seed) and 10 nm PAEs for the fcc phase (shell), chosen due to the pronounced differences in structural symmetry and nanoparticle size would be likely to lead to interesting and observable optical responses (Figure 5a, Figure S11). By employing scattering matrix theory, the refractive index (n) and the extinction coefficient (k) across the visible and near‐infrared (near‐IR) regions were determined for these two phases in each structure (Figure 5b). The bcc phase, composed of the 20 nm PAEs, had a higher refractive index than the fcc phase, composed of the 10 nm PAEs, primarily due to the higher packing density of the nanoparticles in the bcc phase relative to the fcc phase (Table S6).

Notably, when their refractive indices were examined as a function of wavelength, both phases exhibited peaks that were redshifted relative to the localized surface plasmon resonance (LSPRs) of the individual, dispersed gold nanoparticles (Figure 5b). The redshift is due to the influence of interparticle coupling and lattice symmetry on the optical properties of the system. The absorption spectra of the two phases revealed distinct, phase‐dependent behavior in the visible range: the bcc phase composed of 20 nm PAEs exhibited up to 90% absorption in this region, while the fcc phase composed of 10 nm PAEs showed lower overall absorption, featuring pronounced peaks centered at approximately 600 and 720 nm. Both bcc and fcc structures became nearly lossless in the near‐infrared (near‐IR) region (Figure 5c).

The distinct absorption properties of the bcc and the fcc phases enable wavelength‐selective optical waveguiding based on phase heterogeneity. FDTD simulations of a representative heterostructure comprised of an RD bcc core assembled from 20 nm PAEs encapsulated by an octahedron fcc shell comprised of 10 nm PAEs, revealed light absorption at three different wavelengths across the visible and near‐IR spectrum (Figure 5d–f). At 400 nm, the maximum light field absorption happens in the bottom part of the bcc core (Figure 5d). In contrast, at 650 nm (Figure 5e), the peak light absorption shifts to the top section of the bcc core and extends into the surrounding fcc shell. At 720 nm, the fcc phase dominates absorption, while the bcc core becomes effectively transparent, allowing light to pass through without loss and resulting in strong localized absorption within the fcc shell (Figure 5f). This wavelength‐dependent spatial distribution of absorption highlights the potential of these heterostructures for photothermal applications, offering precise spatial and spectral control over light absorption and energy localization within the material.

In conclusion, a universal strategy for the stepwise heteroepitaxial growth of colloidal crystals with different phases is presented (even in the presence of a bcc‐fcc phase misfit as large as 34%). By leveraging the programmable nature of DNA bonding and its inherent flexibility, this approach enables precise control over the formation of complex heterostructures in colloidal crystal systems. Although this study focuses on gold nanoparticle‐based PAEs, it can be extended to DNA‐functionalized nanoparticles with other compositions, as the design rules are independent of particle composition and solely dependent on particle size and shape and DNA sequences [33]. For instance, the gold nanoparticles could be substituted with quantum dots to enable the formation of new functional heterostructures within colloidal crystal systems. Notably, this is the first report of a bcc‐to‐fcc phase transition in colloidal crystal systems, revealing striking structural analogies to epitaxial interfaces in atomic crystals and underscoring the shared fundamental principles governing crystallization across length scales. The lattice mismatches (18%) accommodated through DNA bonding in this study exceed those typically observed in atomic heteroepitaxy, highlighting the great potential of this technique for engineering complicated heterostructures. Furthermore, the demonstration of wavelength‐selective optical waveguiding driven by phase heterogeneity highlights the potential for controlling the spatial distribution of absorbed light within such heterostructures. Overall, this work establishes colloidal crystal engineering with DNA as a versatile and powerful platform for heteroepitaxial growth, opening new pathways for the design of hierarchical metamaterials with tunable structures and optical characteristics.

## Conflicts of Interest

The authors declare no conflicts of interest.

## Supporting information




**Supporting File**: adma73583‐sup‐0001‐SuppMat.docx.

## Data Availability

The data that support the findings of this study are available from the corresponding author upon reasonable request
