# Supplementary material for: Programmable Stepwise Heteroepitaxial Growth of Colloidal Crystals With Different Phases
Source: Adv Mater. 2026 Jun 3;38(38):e73583. doi: 10.1002/adma.73583 (PMC13351763; doi:10.1002/adma.73583)
Supplement: Supplementary file 1 — Supporting File: adma73583‐sup‐0001‐SuppMat.docx. [file ADMA-38-e73583-s001.docx]

Supplementary Information

**Programmable stepwise heteroepitaxial growth of colloidal crystals with different phases**

Xiaowei Liu^1,2^*, Yuanwei Li^2,3^*, Ramin Yazdaanpanah^4^, Ye Zhang^2,5^, Rachel R. Chan^2,5^, Xiaobing Hu^1^, Yiming Yang^2,5^, Vinayak P. Dravid^1,2^, Koray Aydin^2,4^, and Chad A. Mirkin^1,2,3,5^.

1 Department of Materials Science and Engineering, Northwestern University, Evanston, IL 60208

2 International Institute for Nanotechnology, Northwestern University, Evanston, IL 60208

3 Department of Chemical and Biological Engineering, Northwestern University, Evanston, IL 60208

4 Department of Electrical and Computer Engineering, Northwestern University, Evanston, IL 60208

5 Department of Chemistry, Northwestern University, Evanston, IL 60208

(* denotes equal contribution)

**DNA design and synthesis.**

All oligonucleotide strands used for stepwise heteroepitaxial growth were meticulously designed (Table S1) prior to synthesis. The OligoAnalyzer™ Tool from Integrated DNA Technologies was used to calculate the theoretical binding energy and melting temperature for both A-B and B-B hybridization. Phosphoramidites for oligonucleotide synthesis were obtained from Glen Research. Synthesis was carried out on a MerMade 12 automated oligonucleotide synthesizer.

Following synthesis, the oligonucleotides were cleaved from the controlled pore glass beads using a 1:1 mixture of 30% ammonium hydroxide and 40% aqueous methylamine solution, incubated at 55 °C for 30 minutes. The resulting solution was evaporated under a nitrogen gas flow, and the oligonucleotides were further purified using reverse-phase high-performance liquid chromatography (HPLC) with a Varian Microsorb C18 column. The DMT functional groups were removed by treating the purified oligonucleotides with acetic acid and ethyl acetate solutions. Finally, all oligonucleotides were characterized using matrix-assisted laser desorption/ionization time-of-flight mass spectrometry (MALDI-TOF MS) to confirm their molecular mass and purity. UV-Vis spectroscopy was employed to determine oligonucleotide concentrations.

| **DNA Types** | **DNA Sequence (5ʹ to 3ʹ)** |
| --- | --- |
| Anchor A | TCA ACT ATT CCT ACC TAC - (Sp18)_2_ - SH |
| Linker A | GTA GGT AGG AAT AGT TGA- (Sp18) - TAGCTA TCTCT |
| Anchor B | TCC ACT CAT ACT CAG CAA - (Sp18)_2_ - SH |
| Linker B | TTG CTG AGT ATG AGT GGA – (Sp18) - AGAGA TAGCTA |

**Table S1.** **DNA sequences for stepwise heteroepitaxial growth.**

**Nanoparticle functionalization with DNA.**

All gold nanoparticles were purchased from Ted Pella, and their functionalization with DNA followed established literature procedures^1^. Briefly, DNA anchor strands were incubated with 100 mM dithiothreitol (DTT) for 1 hour to cleave the disulfide ends. Excess DTT was removed using a NAP25 column (GE Healthcare). The anchor strands were then added to the corresponding gold nanoparticle suspensions (approximately 10 nmol DNA per ml of the original Au nanoparticle solution from Ted Pella). Sodium dodecyl sulfate (SDS, 1 wt%) and 1 M sodium phosphate (pH 7.5) were added to achieve final concentrations of 0.01 wt% SDS and 10 mM sodium phosphate, respectively. Subsequently, 5 M NaCl (aq) was incrementally added to the Au nanoparticle solutions every 30 minutes to reach final NaCl concentrations of 0.05, 0.1, 0.2, 0.3, 0.4, and 0.5 M. The solutions were briefly sonicated after each addition. After the final NaCl addition, the nanoparticle solutions were placed on a shaker at 1,000 rpm overnight to ensure dense loading of oligonucleotides. Following this incubation, the nanoparticles were centrifuged three times to remove excess DNA. After each centrifugation, the supernatant was discarded and replaced with a solution containing 0.5 M NaCl, 0.01 M sodium phosphate buffer (pH 7.4), and 0.01 wt% SDS. The final solution, referred to as the PAE solution, was used for the assembly of colloidal crystals in the next step.

**UV–Vis Spectroscopy Melting Experiments**

Thermal UV–vis experiments were conducted using a Cary 5000 UV–vis–NIR spectrometer to determine the melting temperatures of assemblies based on our DNA design. The assembly solution, with a total volume of 800 µL, was prepared in quartz cuvettes with magnetic stirring. The solution contained A and B PAE solutions, A and B linker DNA strands, 0.5 M NaCl, 0.01 M sodium phosphate buffer (pH 7.4), and 0.01 wt% SDS. The solution was heated above its melting temperature (typically starting at 65 °C) and subsequently cooled to 25 °C at a rate of 0.1 °C/min. During the slow cooling process, extinction at the localized surface plasmon resonance (LSPR) of the nanoparticle core was measured every minute. The A and B PAE pairs were combined in a 1:7 ratio, with detection carried out at 520 nm.

| **PAE interaction types** | **Annealing temperature** |
| --- | --- |
| ‘A’ PAE-‘B’ PAE complementary interaction | T_a1_ = ~60 °C |
| ‘B’ PAE-‘B’ PAE self-complementary interaction | T_a2_ = ~35 °C |

**Table S2. Annealing temperature of PAE interaction during slow-cooling process (20-nm gold nanoparticle PAEs)**

**Stepwise heteroepitaxial growth of colloidal crystals with bcc and fcc phases**

The stepwise heteroepitaxial growth of colloidal crystals was carried out using a two-pot slow-cooling annealing process with a ProFlex PCR system (Applied Biosystems). In the first step, equal amounts of A and B PAE solutions were added to PCR tubes, followed by the injection and mixing of both linker A and linker B DNA strands with the PAE solutions. The mixture was then subjected to a gradual temperature decrease from 70 °C to 50 °C at a rate of 0.01 °C per minute. The resulting products (bcc seeds) were subsequently transferred and mixed with additional B PAE solutions and linker B DNA strands for the second step. This step involved another slow cooling process, from 50 °C to 25 °C, at the same rate of 0.01 °C per minute. To ensure the complete growth and encapsulation of the bcc seeds by the fcc structures, the amount of B PAE and linker B strands added in the second step was six times greater than that used in the first step.

We also experimentally tested a one-pot slow-cooling method using an initial B:A molar ratio of 7:1. The one-pot approach also produced the targeted heterostructures. However, the current two-pot strategy had a slight advantage over the one-pot approach, yielding final products that were more isolated and exhibited well-defined fcc–bcc heterostructures. One possible explanation is that, in the one-pot approach, all components are introduced into a single reaction environment. Although the A–B and B–B binding strengths are thermodynamically well-separated through DNA sequence design, the excess concentration of B PAEs in solution can still undergo kinetic trapping during nucleation, resulting in the formation of independent fcc crystals. For the two-pot approach, it ensures that the bcc seeds are intentionally established first and then fcc growth occurs preferentially on the bcc seeds rather than through independent nucleation in solution, due to the substantially reduced nucleation energy barrier for heterogeneous growth. The B:A stoichiometry during the second step was optimized by testing molar ratios 3:1, 4:1, 5:1, and 6:1. At the lower ratios of 3:1 and 4:1, the amount of B PAEs in solution was insufficient to fully cover the bcc seeds. In contrast, the higher ratios of 5:1 and 6:1 resulted in the formation of bcc–fcc heterostructures. To ensure that enough B PAEs were present in solution for complete overgrowth, a B:A ratio of 6:1 was used.

**Resin embedding of silica-embedded crystals and cross-section using ultramicrotomy**

The colloidal crystal samples were stabilized using the silica-embedding method, as described in the literature^2^. Subsequently, resin embedding of the silica-embedded colloidal crystals was performed^3^. Briefly, the silica-embedded samples were placed in 10 µL of a 2% low-melt agarose solution to form an agarose gel encapsulating the samples. The agarose gel was then dehydrated by sequential immersion in anhydrous ethanol solutions of increasing concentrations (30%, 50%, 70%, 80%, 90%, and 100%). Following dehydration, the samples in 100% ethanol underwent solvent exchange with acetone twice for 10 minutes each. While in acetone, the agarose-encapsulated samples were embedded in EMBed-812 resin (Electron Microscopy Sciences) according to the manufacturer's standard protocol. The samples were polymerized and solidified at 55 °C for 48 hours, after which the resin was sectioned into 100 nm slices using a Leica EM UC7 ultramicrotome.

**Electron microscopy characterization and phase determination**

Stabilized colloidal crystals were drop-cast onto silicon wafers for scanning electron microscopy (SEM) or onto carbon-coated copper mesh grids (Ted Pella) for transmission electron microscopy (TEM) analyses. SEM characterization of crystal habits was performed using a JEOL JSM-7900FLV microscope with an accelerating voltage of 15 kV, backscattered electron detection, and a working distance of approximately 5 mm. TEM imaging of cross-sections of colloidal crystals was conducted using a Hitachi HD-2300A STEM in TEM mode, operating at an accelerating voltage of 200 kV for Z-contrast imaging. Serial tilting experiments were performed on the JEOL ARM 200CF microscope, operated at 200 kV. The tilt series high-angle annular dark field (HAADF) and annular bright field (ABF) images were collected with a range from −60° to +50° with a 10° increment. For the serial tilting experiment, Hummingbird Scientific’s single-tilt tomography TEM holder was used. The PAE arrangements, spacing, and diffraction patterns from the simulated models were carefully compared with the experimental TEM diffraction patterns to identify the most appropriate projection planes for these regions and determine their index planes and lattice symmetries. The simulated diffraction patterns and models were generated using CrystalMaker, based on lattice parameters obtained from SAXS measurements.

**SAXS data analysis**

The Life Science X-ray Scattering (LiX) beamline at sector 16 (16-ID) of the National Synchrotron Light Source II at Brookhaven National Laboratory was used for all SAXS experiments (X-ray wavelength 0.8 Å from 6-18 keV). Colloidal crystals were sealed in 1.5 mm quartz capillary tubes and exposed to the beam for approximately 1 second to collect the SAXS signals. After collecting SAXS measurements, all peaks were indexed and the lattice constants were calculated using the software package SPACEGROUP (<https://github.com/byeongdu/spacegroup>).

**Colloidal crystal yield after the first and second slow-cooling processes**

The dominant products after the first slow-cooling process are single-phase bcc colloidal crystals, and the dominant products after the second slow-cooling process are overgrown heterostructures (Fig. S2d), with little to no detectable population of standalone bcc or fcc single crystals. Several key driving forces favor heteroepitaxial growth of the fcc phase on a bcc template despite the energetic penalty associated with lattice mismatch. First, according to classical heterogeneous nucleation theory, the nucleation barrier is significantly lower at a pre-existing interface than in the bulk solution. Although lattice mismatch introduces an additional energetic penalty, a pre-existing bcc template reduces a substantial portion of the free-energy barrier required for fcc nucleation. As a result, the net effect is that the fcc-on-bcc heterostructure forms. Secondly, during the second slow-cooling step, B PAEs preferentially bind to the exposed A PAEs on the surface because the A–B interaction is stronger than the B–B interaction. This preferential A–B hybridization promotes nucleation and growth of the fcc phase on bcc templates, rather than the formation of independently nucleated fcc crystals in the bulk solution. Finally, the DNA shells on the particle surfaces are soft and deformable, so the bcc/fcc mismatch is not borne entirely as hard-core lattice strain. Instead, part of the mismatch is accommodated by local compression, stretching, and angular rearrangement of the DNA brushes, which lowers the energy generated by lattice mismatch of fcc and bcc. To optimize the stoichiometry of the two types of PAEs in the second step, we tested B:A molar ratios 3:1, 4:1, 5:1, and 6:1 and collected SEM images under each condition. At the lower ratios of 3:1 and 4:1, the amount of B PAEs in solution was insufficient to fully cover the bcc seeds. In contrast, the higher ratios of 5:1 and 6:1 resulted in the formation of bcc–fcc heterostructures. To ensure that enough B PAEs were present in solution for complete overgrowth, a B:A ratio of 6:1 was used.

**Colloidal crystal size distributions after the first and second slow-cooling processes**

The rhombic dodecahedron crystals in the SEM images exhibit projected sizes in the range of approximately 1.3–2.7 µm, with a mean size of 1.82 ± 0.42 µm (n = 12, mean ± standard deviation, equivalent size) after the first slow cooling process (Table S3). The overgrown heterostructure crystal size measured from SEM image projections was 13.2 ± 2.3 µm (n = 12, mean ± standard deviation, equivalent size) with a range of 9.5–16.7 µm, after the second cooling process (Table S4).

**Table S3. Colloidal crystal size after the first slow-cooling process**

| **Crystal index** | **Long axis (µm)** | **Short axis (µm)** | **Equivalent size (µm)** |
| --- | --- | --- | --- |
| 1 | 2.92 | 2.48 | 2.69 |
| 2 | 2.58 | 2.31 | 2.44 |
| 3 | 2.40 | 2.00 | 2.19 |
| 4 | 2.03 | 1.85 | 1.94 |
| 5 | 1.95 | 1.68 | 1.81 |
| 6 | 1.82 | 1.68 | 1.75 |
| 7 | 1.80 | 1.58 | 1.69 |
| 8 | 1.72 | 1.41 | 1.56 |
| 9 | 1.64 | 1.49 | 1.56 |
| 10 | 1.63 | 1.38 | 1.50 |
| 11 | 1.49 | 1.33 | 1.41 |
| 12 | 1.41 | 1.22 | 1.31 |

**Table S4. Colloidal crystal size after the second slow-cooling process**

| **Crystal index** | **Long axis (µm)** | **Short axis (µm)** | **Equivalent size (µm)** |
| --- | --- | --- | --- |
| 1 | 12.6 | 12.2 | 10.3 |
| 2 | 17.0 | 11.9 | 11.9 |
| 3 | 17.8 | 13.4 | 14.3 |
| 4 | 17.8 | 11.0 | 11.3 |
| 5 | 21.9 | 15.7 | 16.5 |
| 6 | 14.5 | 7.6 | 9.5 |
| 7 | 18.7 | 15.0 | 14.1 |
| 8 | 18.9 | 12.9 | 13.6 |
| 9 | 26.3 | 14.7 | 16.7 |
| 10 | 16.7 | 11.7 | 12.8 |
| 11 | 19.3 | 18.0 | 15.2 |
| 12 | 20.8 | 12.3 | 13.8 |

**A simplified model for PAE–PAE interactions mediated by DNA brushes**

We modeled each programmable atom equivalent (PAE) as a hard nanoparticle core of radius $R$surrounded by a DNA shell of total thickness $h=b+s$, where $b$ is the length of the non-sticky DNA brush and $s$ is the length of the sticky-end. At a transition interface between two PAE superlattices with lattice spacings $a_{1}$ and $a_{2}$, the lattice mismatch is defined as $f={(a}_{2}- a_{1})/a_{1}$. This mismatch is primarily accommodated through elastic deformation of the non-sticky DNA brush rather than through defect formation within the crystal lattice. The characteristic lateral displacement required to maintain registry across the interface scales as $\delta_{t}=fa$, where $a=2(R+h)$is the center-to-center spacing of neighboring PAEs. Because the DNA shell is curved, this lateral distortion induces a normal displacement of order $\delta_{n}=\delta_{t}^{2}/a$. Using an effective harmonic description for the DNA brush, the interfacial strain energy per bonded site can therefore be written as

$U_{\mathrm{strain}}\left( f \right)=\frac{1}{2}K_{t}\delta_{t}^{2}+\frac{1}{2}K_{n}\delta_{n}^{2}$ Equation (S1)

For this equation, we assumed that the lattice strain energy associated with the lattice mismatch $f$ is contributed by both the lateral and normal deformation of the DNA brushes, where $K_{t}$ and $K_{n}$ represent the effective lateral and normal spring constants of the DNA brush-coated interface, respectively. For a simplified entropic elasticity model, these stiffnesses scale as

$K_{t}=\chi N_{c}\frac{3K_{B}T}{L_{P}b}$ Equation (S2)

$K_{n}=\eta N_{c}\frac{3K_{B}T}{L_{P}b}$ Equation (S3)

where $N_{c}$ is the number of DNA chains that effectively share the interfacial load, $L_{P}$ is the persistence length of the non-sticky DNA segment, $K_{B}$ is the Boltzmann constant, T is absolute temperature, and $\chi$ and $\eta$ are geometric factors describing lateral and normal loading, respectively. Coherent heteroepitaxial growth is expected when the elastic penalty remains smaller than the cohesive free energy per interfacial contact, $U_{\mathrm{coh}}$ supplied by multivalent DNA hybridization

$U_{\mathrm{strain}}\left( f \right)\leq U_{\mathrm{coh}}$ Equation (S4)

Considering the critical case for lattice strain

$U_{\mathrm{strain}}\left( f \right)=\frac{1}{2}K_{t}\delta_{t}^{2}+\frac{1}{2}K_{n}\delta_{n}^{2}=\frac{1}{2}{\chi N}_{c}\frac{3K_{B}T}{L_{P}b}{(fa)}^{2}+\frac{1}{2}\eta N_{c}\frac{3K_{B}T}{L_{P}b}{(f^{2}a)}^{2}=U_{\mathrm{coh}}$ Equation (S5)

This equation highlights two important points. First, when the size of PAE is constrained ($a$), a longer non-sticky brush ($b$), corresponding to a lower effective interfacial stiffness ($L_{P}$), should enable the accommodation of larger lattice mismatch. Second, stronger sticky-end hybridization should increase the cohesive interfacial energy ($U_{\mathrm{coh}}$), thereby allowing the system to tolerate greater lattice mismatch without losing coherence. These considerations help explain why the DNA design used in this work can accommodate an exceptionally large lattice, reaching up to 18%. This high tolerance is consistent with the combination of highly flexible DNA chains, arising from the three spacer-18 segments from non-sticky regions, and relatively strong 11-bp DNA hybridization interactions.

**Table S5. Interplanar particle spacing for (110)_bcc_//****(111)_fcc_ transition interface (**$\text{a}_{\text{fcc}}\text{ = 56.6 nm,}\text{ }\text{a}_{\text{fcc}}\text{ = 40.0 nm}$**)**

| **Plane** | **Interplanar spacing** |
| --- | --- |
| ${(1-10)}_{fcc}$ | $d_{{(1-10)}_{fcc}}={a_{fcc}}/\sqrt{2}$ = 40.0 nm |
| ${(11-2)}_{fcc}$ | $d_{{(11-2)}_{fcc}}={a_{fcc}}/\sqrt{6}$ = 23.1nm |
| ${(001)}_{bcc}$ | $d_{{(001)}_{bcc}}=a_{bcc}$ = 40.0 nm |
| ${(1-10)}_{bcc}$ | $d_{{(1-10)}_{bcc}}={a_{bcc}}/\sqrt{2}$ = 28.3 nm |

**
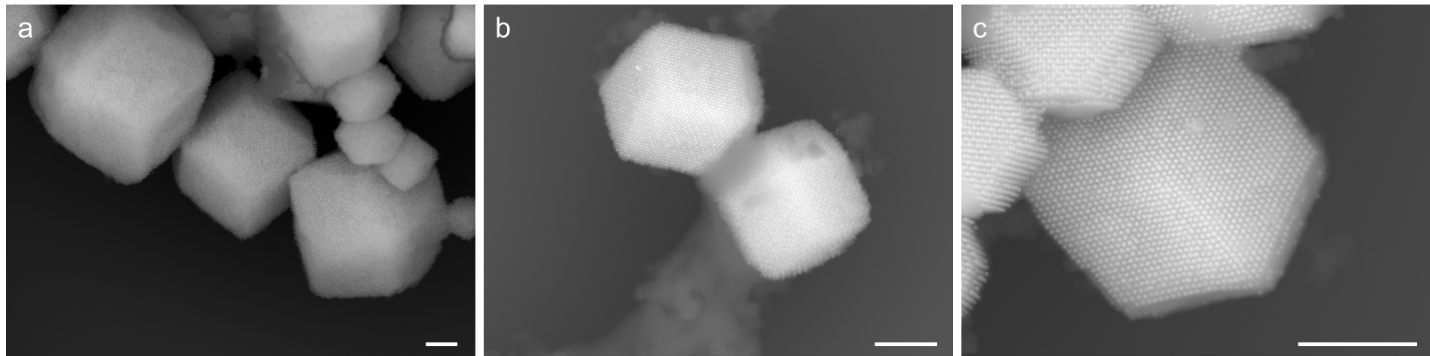
**

**Figure S1. The 1^st^ step products with rhombic dodecahedron crystal habits with bcc structure.** Scale bars, 500 nm.


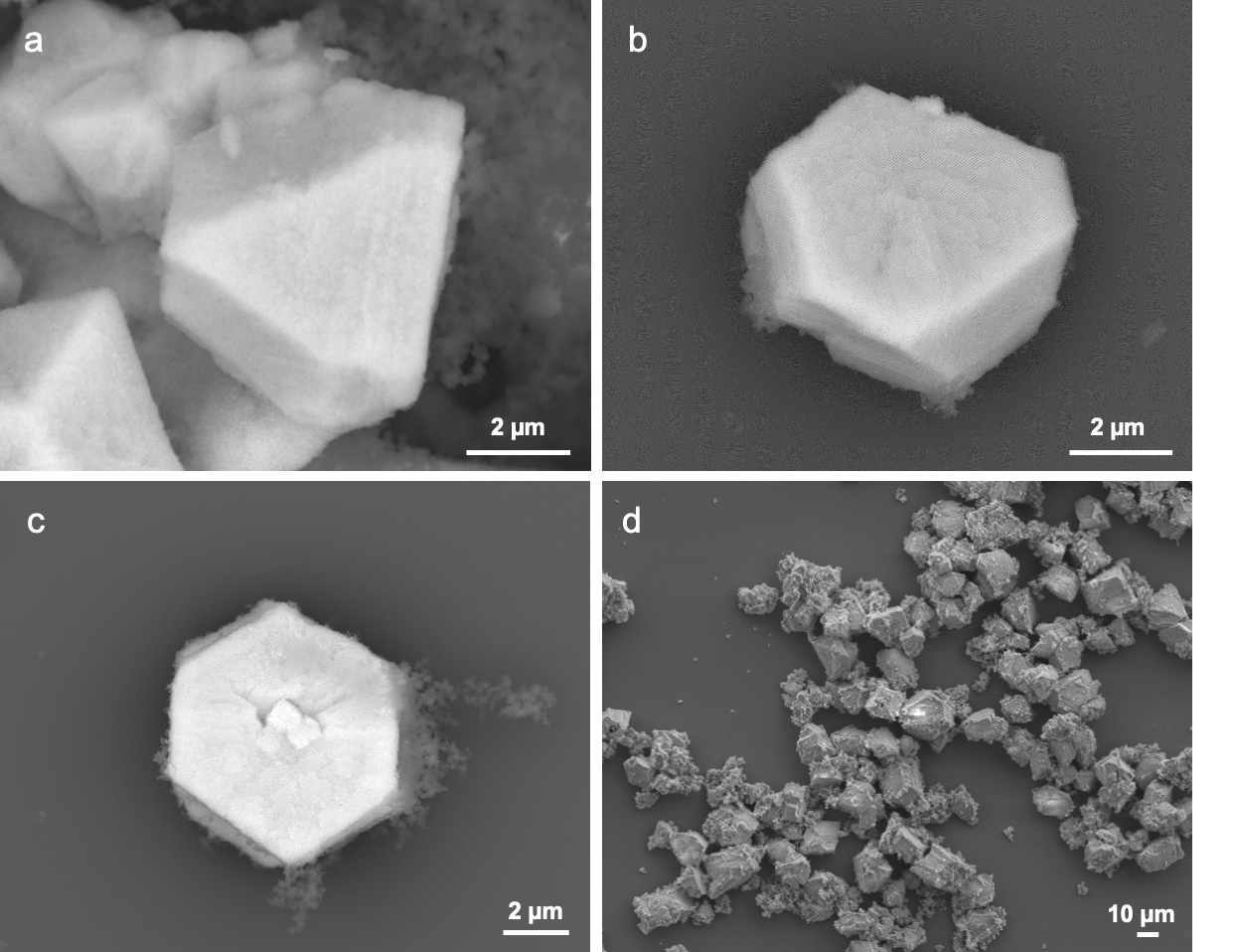
**Figure S2. The** **2^nd^ step products with octahedron or truncated octahedron crystal habits with fcc structure.**


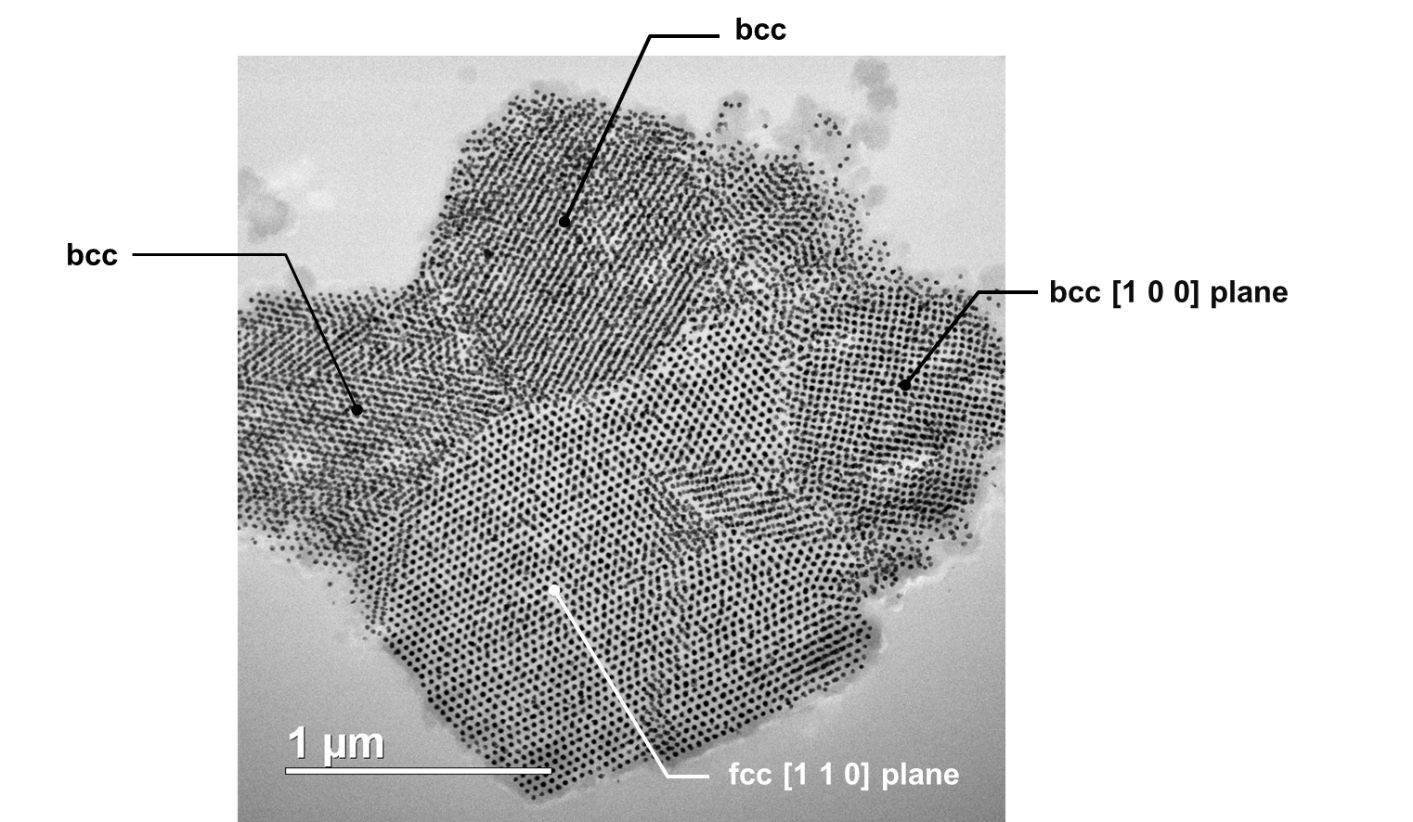


**Figure S3. The cross-section ABF images of 2nd step products with octahedron or truncated octahedron crystal habits.** Scale bars, 200 nm.


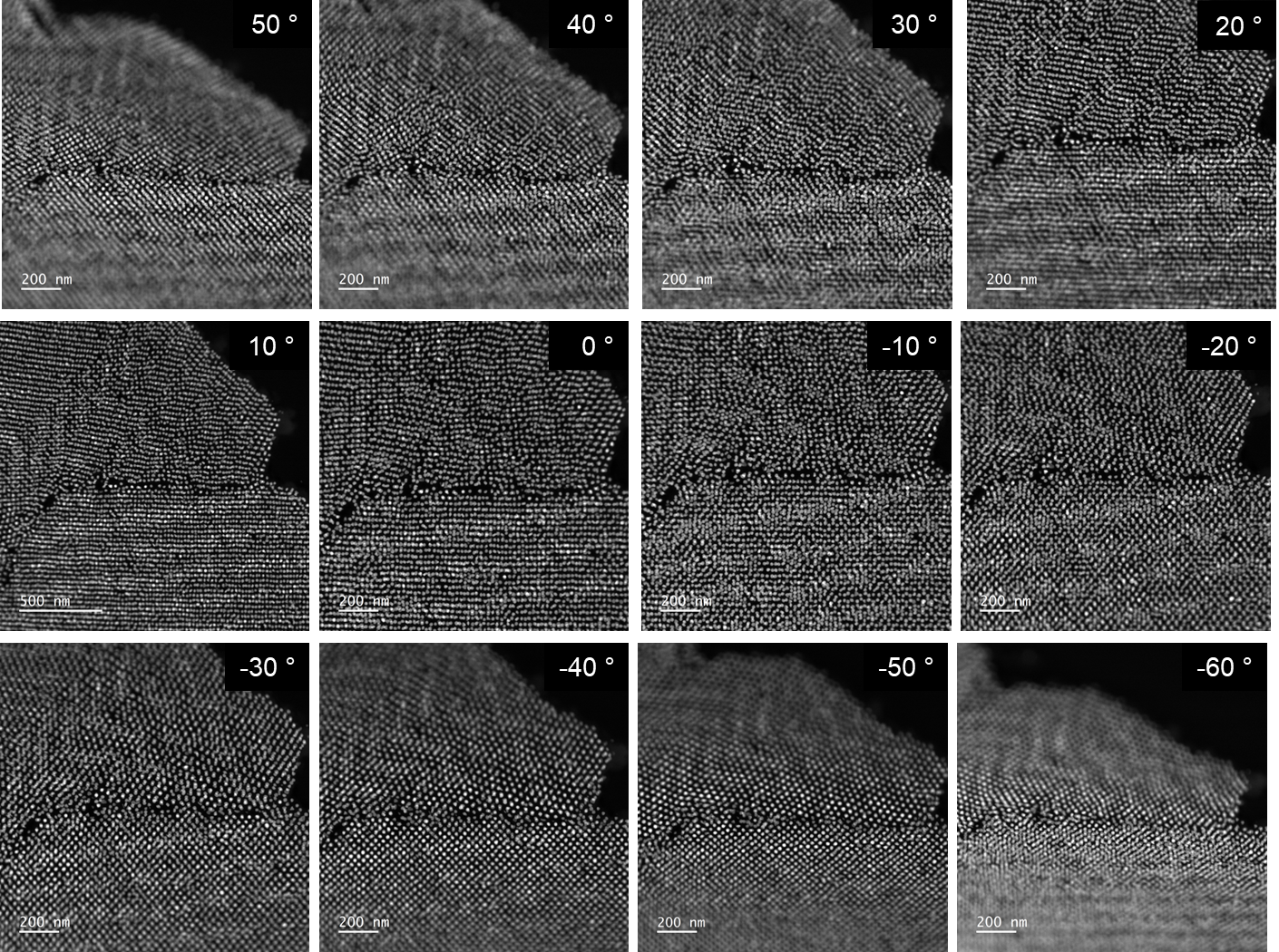


**Figure S4. Serial HAADF images obtained at different tilting angles showing the features of transition interface between the bcc and fcc structure.** The interval serial tilt is 10°.

**
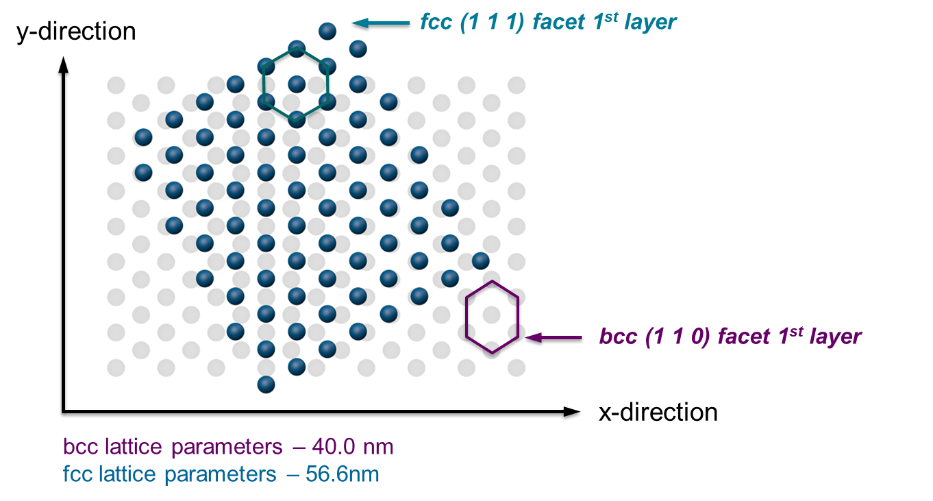
**

**Figure S5. Schematic of theoretical lattice mismatch at transition interface between bcc and fcc structure.**

**
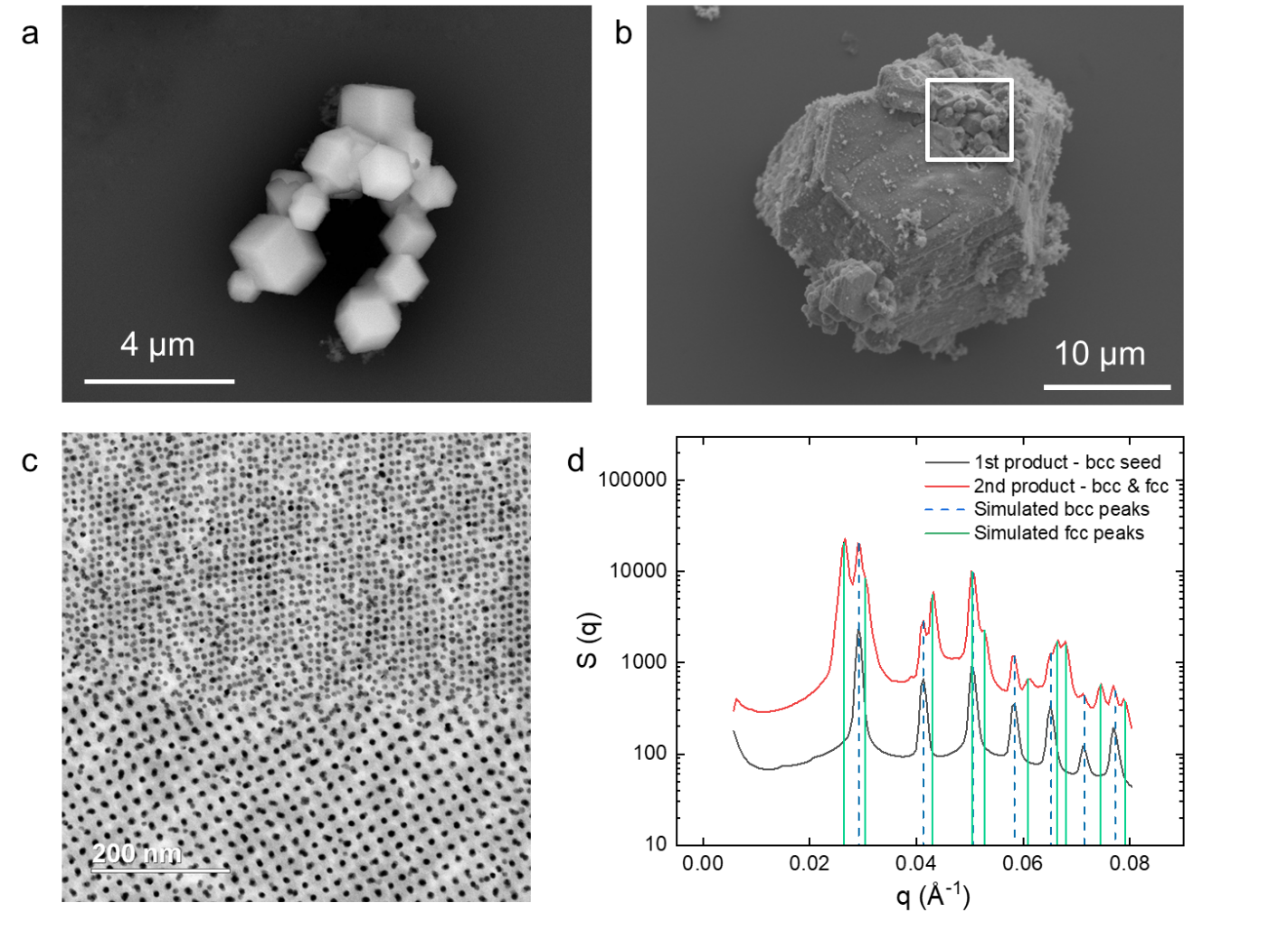
**

**Figure S6. Heteroepitaxial growth of the fcc on the bcc seeds using 10 nm PAEs.** a, SEM images of the bcc seeds with rhombic dodecahedral (RD) crystal habits. b, SEM images of fcc crystals grown on the cluster of bcc seeds. c, ABF image of the transition interface between bcc and fcc phases in a 10 nm PAE system. d, The SAXS signal for 1st product shows bcc symmetries while the one for 2nd product exhibits the combination of fcc and bcc symmetries for 10 nm PAEs.


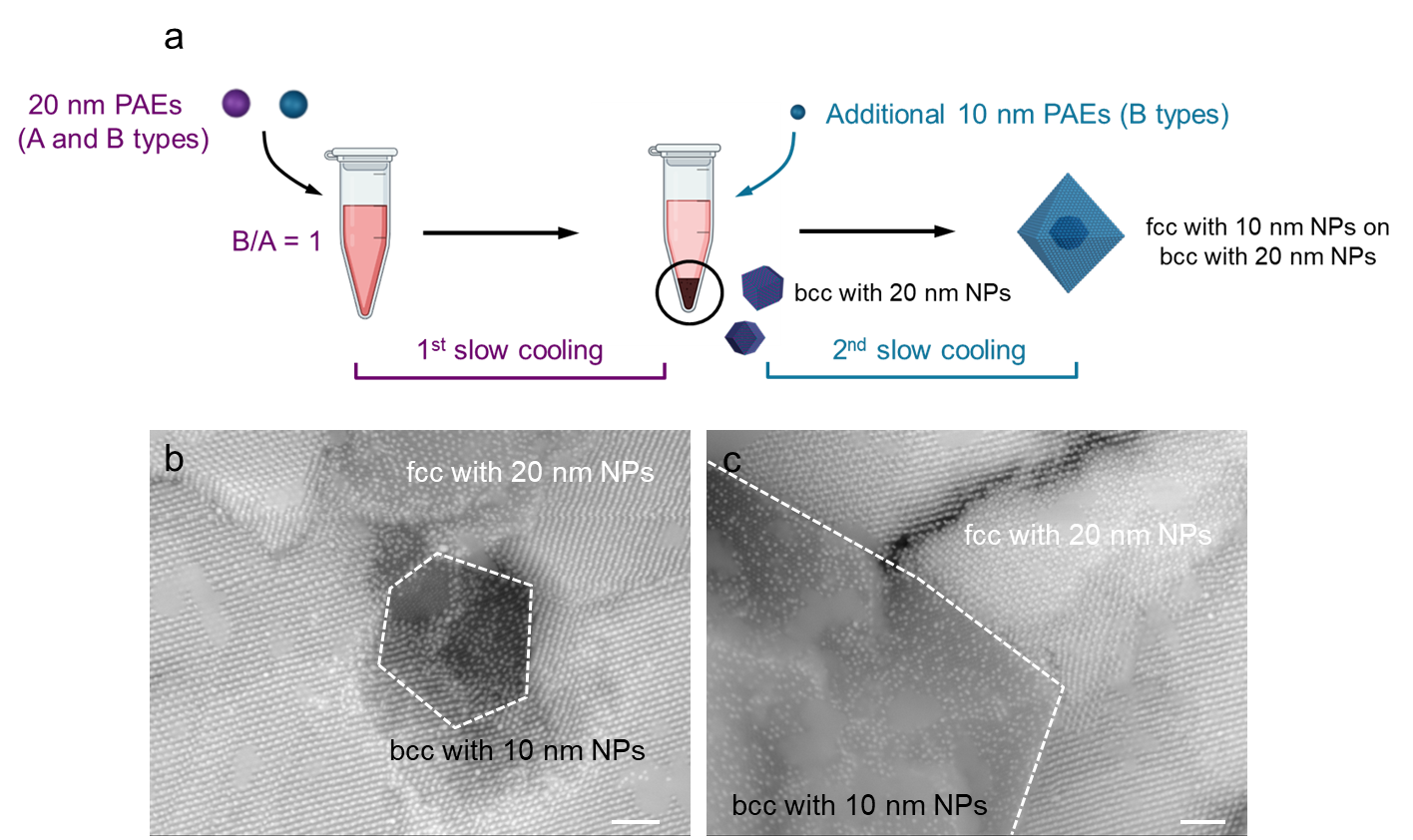


**Figure S7. Heteroepitaxial growth of the fcc phase with 20 nm PAEs on the bcc seed with 10 nm PAEs.** a, The synthesis scheme of heteroepitaxial growth. b,c, SEM micrographs obtained using the backscattering electrons. The brighter and bigger nanoparticles are 20 nm PAEs and the darker and smaller ones are 10 nm PAEs. Scale bars, 2 µm.


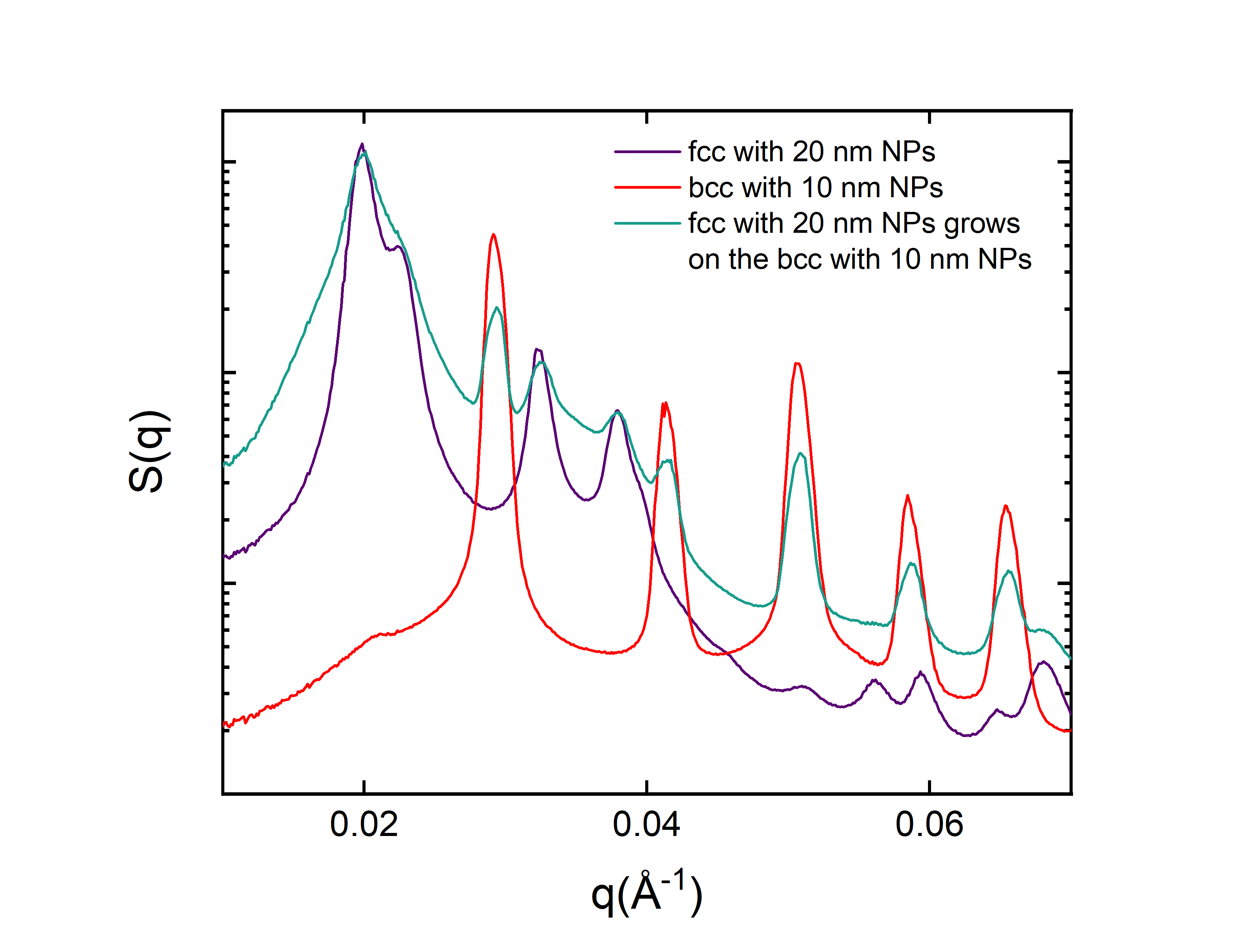


**Figure S8. SAXS characterization of heteroepitaxial growth of the fcc phase with 20 nm PAEs on the bcc seed with 10 nm PAEs, compared to the corresponding single phases.**


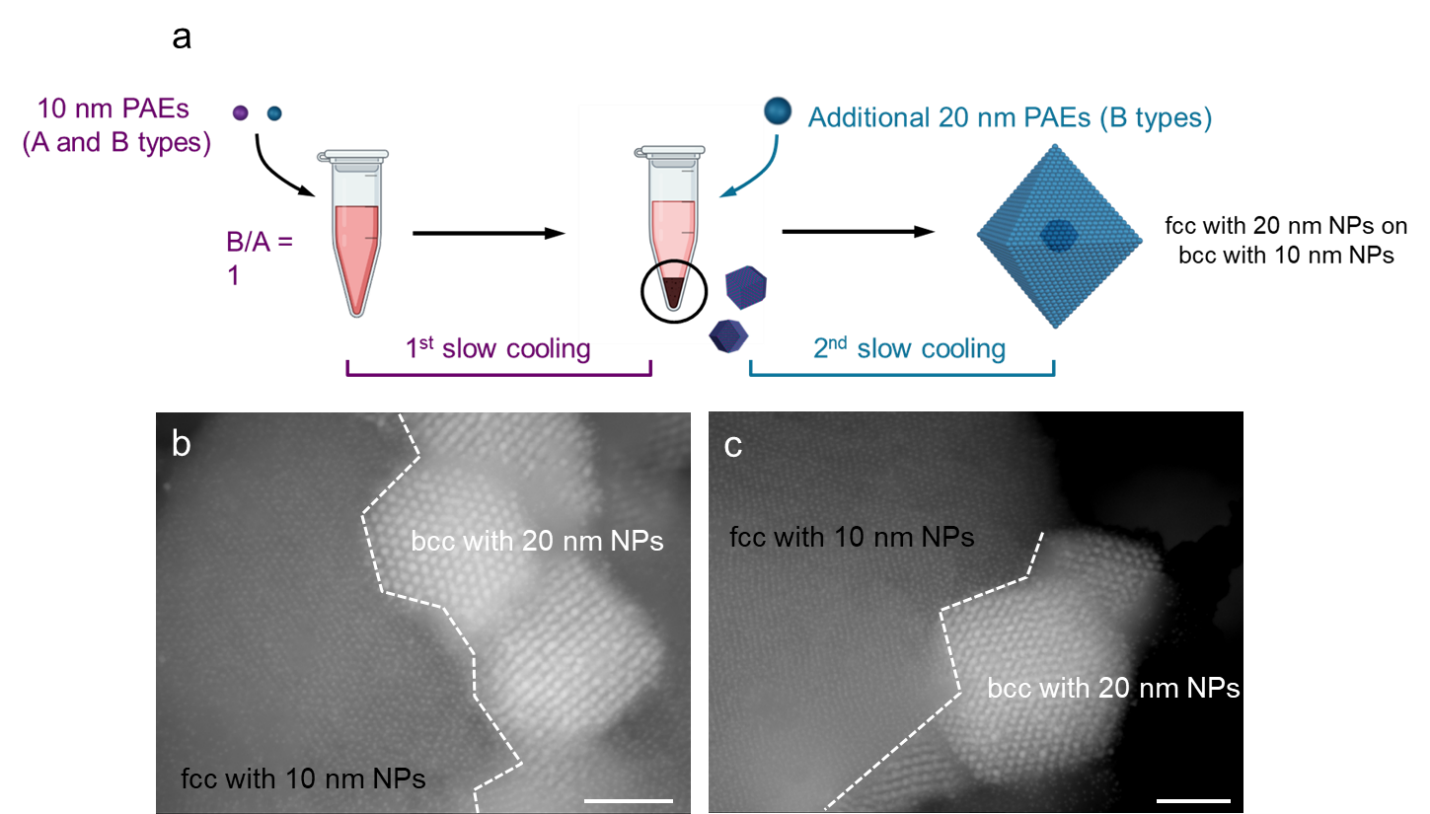


**Figure S9. Heteroepitaxial growth of the fcc phase with 10 nm PAEs on the bcc seed with 20 nm PAEs.** a, The synthesis scheme of heteroepitaxial growth. b,c, SEM micrographs imaged using backscattering electrons. The brighter and bigger nanoparticles are 20 nm PAEs and the darker and smaller ones are 10 nm PAEs. Scale bars, 2 µm.

**
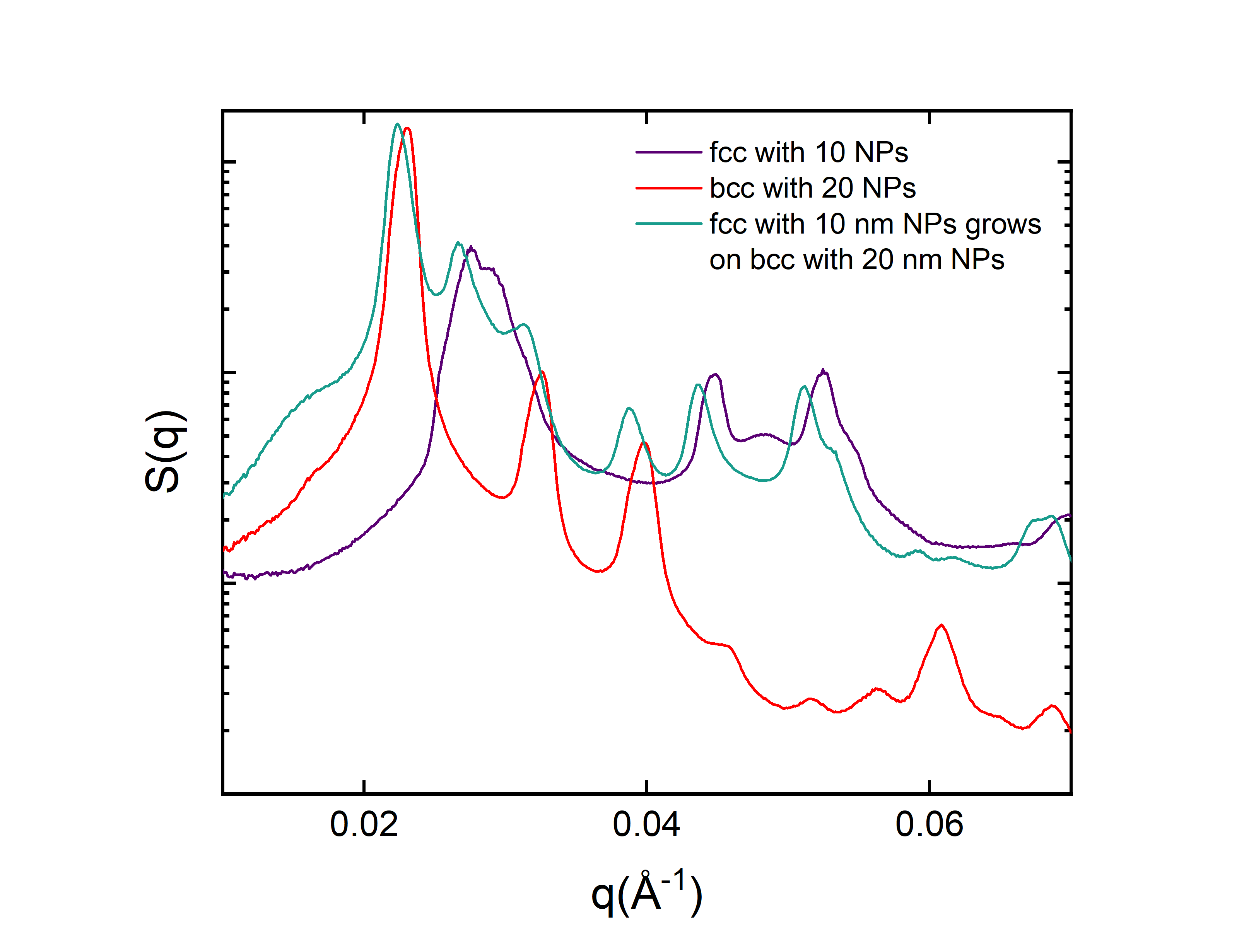
**

**Figure S10. SAXS characterization of heteroepitaxial growth of the fcc phase with 10 nm PAEs on the bcc seed with 20 nm PAEs, compared to the corresponding single phases.**

**Optical simulation for heteroepitaxial-grown colloidal crystals**

We used Lumerical software, a commercial Maxwell equation solver based on the finite-difference time-domain (FDTD) method, to calculate the scattering matrix of each bcc and fcc structure with 10 nm and 20 nm nanoparticles. After extracting complex parameters (S-parameters) from an electromagnetic simulation, the effective index can be calculated through an iterative process as:

$$n_{eff}=\frac{1}{kd}\cos^{-1} \left( \frac{1-S_{11}^{2}+S_{21}^{2}}{{2S}_{21}} \right)$$

Where k and d correspond to the wavevector and the thickness of the structure, respectively^4^. In this equation, the system is considered symmetric in the direction of light propagation, and as a result, S_11_ = S_22_ and S_21_ = S_12_. In our simulations, we used the Johnson and Christy dataset for the refractive index of gold nanoparticles^5^. The lattice constant of each structure is extracted from the experimental data. To better realize the refractive index of the effective medium, we assumed the crystals to be infinitely periodic on the plane normal to the light propagation (x-y). Also, as we increase the number of unit cells in the z-direction (direction of light propagation), the accuracy of the effective refractive index data will be increased. Therefore, we considered 10-unit cell layers in the z-direction for bcc and fcc structures to calculate the scattering matrix and effective index data. This number of unit cells provides good accuracy, and it is also computationally cost-efficient. Considering all of these parameters, we implemented periodic boundary conditions in our simulations in x-y directions and a perfectly matched layer (PML) in z-direction.  It is worth mentioning that all of the simulations are done with maximum mesh accuracy, and the minimum allowable mesh size in the system was 0.25 nm.

Figure S11 shows the refractive index and extinction coefficients for the bcc and fcc structures with 10 and 20 nm nanoparticles. In our calculations, the nanoparticles are assumed to be embedded in silica (n=1.42). The fraction filling factor for each of these crystals is also calculated. The result for the filing factor is presented in the table below. The fraction filling factor for each unit cell is calculated by dividing the volume of the unit cell filled with nanoparticles by the total volume of the unit cell. These data correspond to the optical constant data we extracted using the scattering matrix theory. The higher the filling factor, the higher the refractive index. In the table below, in the first row, the specification of each fcc and bcc crystal (size of nanoparticles and the lattice constant for each of them) is mentioned, and in the second row, the filling factor for each crystal is written.

After extracting the optical constants, we used the refractive index of the effective medium to configure the heterostructure and study the total field absorption within the heterostructure in different wavelengths, as presented in Figure 5 of the paper. To calculate the total field absorption, based on experimental data, we considered that the bcc seed has a rhombic dodecahedron shape, and the outside fcc layer has an octahedron orientation. After configuring the heterostructure, we set PML boundary conditions for all x-y-z directions and put a plane wave source in the x-y plane (propagation in z-direction). Then, using the power-absorbed analysis group in Lumerical software, we calculate the total field absorption in the middle of the heterostructure in the x-z plane.

| Symmetry | fcc | bcc | fcc | bcc |
| --- | --- | --- | --- | --- |
| **Nanoparticle sizes** | 10 nm | 10 nm | 20 nm | 20 nm |
| **Lattice constants** | 41.3 nm | 30.5 nm | 56.6 nm | 40 nm |
| **Packing factors** | 0.03 | 0.037 | 0.093 | 0.131 |

**Table S6. The lattice parameters of different phases constructed from nanoparticles of varying sizes and symmetries.**

**Figure S11. Simulated effective index for four different phases in the heteroepitaxial growth**


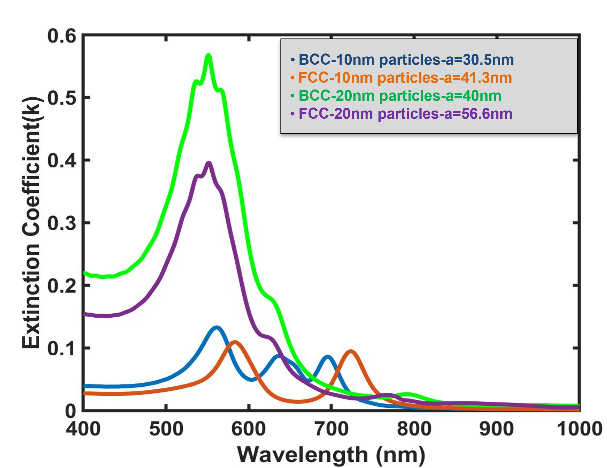

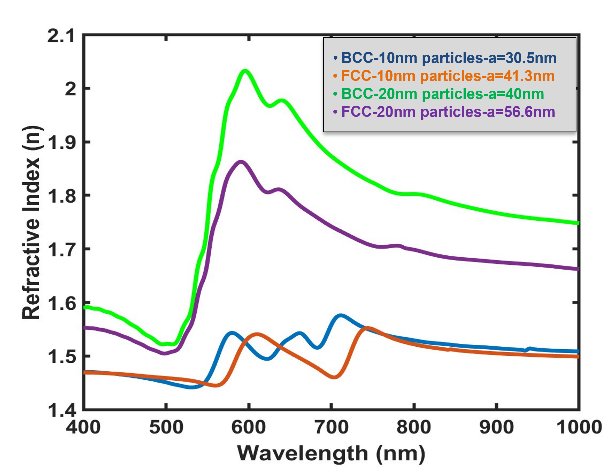


**References**

(1) Macfarlane, R. J.; Lee, B.; Jones, M. R.; Harris, N.; Schatz, G. C.; Mirkin, C. A. “Nanoparticle Superlattice Engineering with DNA.” *Science* **2011**, *334* (6053), 204–208. https://doi.org/10.1126/science.1210493.

(2) Oh, T.; Park, S. S.; Mirkin, C. A.; Oh, T.; Mirkin, C. A.; Park, S. S. “Stabilization of Colloidal Crystals Engineered with DNA.” *Adv. Mater.* **2019**, *31* (1), 1805480. https://doi.org/10.1002/adma.201805480.

(3) Li, Y.; Zhou, W.; Tanriover, I.; Hadibrata, W.; Partridge, B. E.; Lin, H.; Hu, X.; Lee, B.; Liu, J.; Dravid, V. P.; Aydin, K.; Mirkin, C. A. “Open-Channel Metal Particle Superlattices.” *Nature* **2022**, *611* (7937), 695–701. https://doi.org/10.1038/s41586-022-05291-y.

(4) Smith, D. R.; Vier, D. C.; Koschny, T.; Soukoulis, C. M. “Electromagnetic Parameter Retrieval from Inhomogeneous Metamaterials.” *Phys. Rev. E Stat. Nonlin. Soft Matter Phys.* **2005**, *71* (3). https://doi.org/10.1103/physreve.71.036617.

(5) Johnson, P. B.; Christy, R. W. “Optical Constants of the Noble Metals.” *Phys. Rev. B* **1972**, *6* (12), 4370–4379. https://doi.org/10.1103/physrevb.6.4370.
